# Real-world outcomes after switching to faricimab in treatment-resistant diabetic macular edema: A 1-year observational study with choroidal thickness assessment

**DOI:** 10.1371/journal.pone.0351168

**Published:** 2026-06-10

**Authors:** Ryo Tomemori, Hidetsugu Mori, Mari Tanaka, Tatsunori Kiriishi, Masatoshi Omi, Yuki Hattori, Shimpei Oba, Masayuki Ohnaka, Hisanori Imai

**Affiliations:** Department of Ophthalmology, Kansai Medical University, Hirakata, Osaka, Japan; Tsukazaki Hospital, JAPAN

## Abstract

Approximately 40% of diabetic macular edema (DME) cases are resistant to conventional anti-vascular endothelial growth factor (VEGF) therapy. Although faricimab (an antibody against VEGF-A and angiopoietin-2 [Ang-2]) has shown efficacy in treatment-naïve DME, real-world evidence on switching strategies in resistant cases remains limited. We evaluated the 1‑year real‑world effectiveness of switching to faricimab in resistant DME and explored central choroidal thickness (CCT) changes and their relationship to retinal and choroidal responses. This retrospective study included 28 eyes from 19 patients with treatment-resistant DME who switched from aflibercept or ranibizumab to faricimab between May 2022 and August 2023. Central retinal thickness (CRT), CCT, best-corrected visual acuity (BCVA), and injection frequency were evaluated 1 year before, immediately before, and 1 year post-switching. Mean CRT significantly decreased from pre-switching levels of 453 ± 112 μm to 322 ± 60 μm 1-year post-switching (28.9% reduction). The dry macula rate (CRT ≤ 325 μm) was achieved in 53.6% of eyes at 1 year. Injection frequency was significantly higher in the year after switching than in the year before switching (3.5 vs. 5 injections). LogMAR BCVA showed no significant change (0.31 ± 0.27 vs. 0.34 ± 0.32). Ellipsoid zone (EZ)/external limiting membrane (ELM) disruption increased from 28.6% at 1 year before switching to 35.7% immediately before switching, with minimal progression to 39.3% at 1 year post-switching. Mean CCT demonstrated no significant change (238 ± 82 μm vs. 230 ± 62 μm). Faricimab achieved significant anatomical improvements in resistant DME supporting its efficacy in real-world practice. Stable CCT may indicate preserved choroidal perfusion and represent a favorable characteristic of dual VEGF-A and Ang-2 inhibition. The potential suppression of the outer retinal structural deterioration may represent a marker of vascular health during chronic anti-VEGF therapy.

## Introduction

Diabetic macular edema (DME) is a leading cause of vision loss among working-age adults with diabetes [1]. It results from the breakdown of the blood–retinal barrier and increased vascular permeability, primarily driven by vascular endothelial growth factor-A (VEGF-A) [2]. Intravitreal anti-VEGF therapy is the established first-line treatment for DME and has demonstrated substantial benefits in improving visual acuity and reducing macular thickness in both randomized controlled trials and real-world settings [3]. Nevertheless, approximately 40% of patients exhibit suboptimal or resistant responses, characterized by persistent macular edema despite regular anti-VEGF injections [4,5].

The mechanisms underlying treatment resistance in DME are complex and not yet fully understood. Proposed contributors include chronic inflammation, structural alterations in the retinal vasculature, tachyphylaxis to anti-VEGF agents, and activation of VEGF-independent pathways [6,7]. The angiopoietin–Tie2 signaling pathway is a particularly important VEGF-independent mechanism associated with vascular instability and fluid leakage in diabetic retinopathy [8,9]. Angiopoietin-2 (Ang-2), a Tie2 receptor antagonist, is upregulated in diabetic retinopathy and promotes vascular permeability, inflammation, and neovascularization [9]. Elevated levels of Ang-2 in the aqueous humor have been linked to poor response to anti-VEGF monotherapy, suggesting that dual inhibition of both VEGF-A and Ang-2 may offer improved outcomes in treatment-resistant cases [10].

Faricimab is a novel bispecific antibody engineered using CrossMab technology to inhibit both VEGF-A and Ang-2 [11]. In the phase 3 YOSEMITE and RHINE trials, faricimab produced visual outcomes non-inferior to those of aflibercept in treatment-naïve DME, while enabling extended dosing intervals in many patients [12]. Although these trials established faricimab as an effective first-line option, their populations were predominantly treatment-naïve, and real-world evidence regarding its effectiveness as a switch therapy in resistant DME remains limited [13–16].

Recent real-world studies have begun to fill this gap. Pichi et al. reported that poor responders to aflibercept experienced meaningful improvements in best‑corrected visual acuity (BCVA) (+5 letters, P = 0.007) and reductions in central retinal thickness (CRT) (−67.9 μm, P = 0.004) after switching to faricimab [13]. Huber et al. observed significant anatomical improvement at 12 weeks post-switching in aflibercept responders (54%) and non-responders (25%) [14]. Tatsumi et al. found that multiple faricimab injections (≥3) were required for optimal outcomes, with a significant CRT reduction observed by the third injection [15]. Rush further noted that 82.4% of patients with ≥50 μm CRT reduction following the first three faricimab injections were able to maintain ≥8-week treatment intervals with a fluid-free macula at 12 months [16]. Despite these anatomical improvements, visual acuity gains remained modest, likely because of preexisting structural damage in chronically edematous retinas.

Although the anti-VEGF therapy has been extensively evaluated with respect to retinal thickness and function, its effects on the choroidal vasculature remain insufficiently understood. The choroid provides essential metabolic support to the outer retina and the retinal pigment epithelium (RPE), and choroidal dysfunction has been implicated in the pathogenesis of diabetic retinopathy [17,18]. While macular edema and increased retinal thickness are hallmarks of DME, the presence of CCT changes remains controversial [18–21]. Studies assessing CCT during anti-VEGF therapy have yielded inconsistent findings [22–24]. Some have suggested that choroidal thinning may indicate impaired vascular perfusion rather than simple resolution of congestion, raising concerns about potential adverse effects on choroidal circulation [25]. Moreover, Saima et al. recently demonstrated that switching from aflibercept to faricimab led to decreased ocular blood flow in the optic disc region, with a significant correlation between decreased blood flow and decreased macular thickness [26]. However, anatomical CCT changes specifically associated with faricimab therapy have not been systematically examined.

This study therefore had two primary objectives. First, we aimed to validate the real-world efficacy of switching to faricimab in treatment-resistant DME by assessing anatomical outcomes, visual acuity changes, and treatment burden over 1 year. Second, as an exploratory endpoint, we aimed to evaluate alterations in CCT during faricimab therapy and examine the relationship between retinal and choroidal responses. We hypothesized that faricimab would reduce persistent macular edema in treatment-resistant cases and that its dual mechanism could help preserve choroidal vasculature.

## Materials and methods

### Study design and ethical approval

This retrospective observational study was conducted at Kansai Medical University Hospital at the Department of Ophthalmology, in accordance with the Declaration of Helsinki. The study protocol was approved by the Institutional Review Board of Kansai Medical University (approval number: 2024101). Because the study used de‑identified clinical data, the requirement for written informed consent was waived. All patient information was anonymized before the analysis.

### Patient selection

Medical records of all patients with DME who were switched from conventional anti-VEGF therapy (aflibercept or ranibizumab) to faricimab between May 2022 and August 2023 were reviewed. Inclusion criteria were as follows: (1) center-involving DME confirmed by optical coherence tomography (OCT); (2) treatment resistance, defined as CRT ≥ 325 μm despite anti-VEGF injections (aflibercept or ranibizumab) during the 12 months preceding the switching; (3) availability of OCT data at three time points: 1 year before switching, immediately before switching (within 4 weeks), and 1 year post-switching (within 4 weeks of the 12-month follow‑up); and (4) a minimum follow-up period of 12 months after switching to faricimab.

Exclusion criteria were as follows: (1) coexisting retinal diseases that could influence macular thickness or visual acuity, including age-related macular degeneration, retinal vein occlusion, or epiretinal membrane requiring treatment; (2) history of pars plana vitrectomy; (3) history of pan-retinal photocoagulation within 6 months before or post-switching; (4) media opacity compromising OCT imaging quality; and (5) concurrent systemic anti‑VEGF therapy during the study period.

### Treatment protocol

All patients received intravitreal injections of faricimab (6.0 mg in 0.05 mL, Vabysmo; Genentech Inc., South San Francisco, CA, USA) according to a 1 + pro re nata (PRN) regimen. The initial injection was administered at the time of switching. Patients were evaluated monthly, and additional injections were administered when CRT exceeded 325 μm with intraretinal or subretinal fluid, or when CRT increased by more than 50 μm from the previous visit. All injections were administered under sterile conditions in a room setting using standard sterile preparations and topical anesthesia.

### OCT imaging and measurements

Spectral-domain OCT imaging was conducted using the Heidelberg Spectralis OCT system (Heidelberg Engineering, Heidelberg, Germany) at all predefined time points. A standardized protocol was used, including a horizontal raster scan centered on the fovea with a 30° × 30° field of view, and an average additive number of approximately 40 sheets was used. Automatic real-time eye tracking and additive averaging were used to ensure image quality, while the vascular matching–based follow-up function enabled repeated assessments at the same retinal location.

CRT was defined as the mean retinal thickness within a 1 mm diameter circle at the center of the retinal thickness color map, automatically calculated using Heidelberg Spectralis software (software version 6.16.8) from 31 OCT B-scans with an average additive number of approximately 15 sheets and a field of view of 30° × 25°.

CCT was measured manually as the perpendicular distance from the outer border of the RPE to the inner border of the sclera at the subfoveal point on enhanced-depth OCT scans. All measurements were performed by two independent masked graders, and mean values were used for analysis.

Outer retinal integrity was assessed by examining the ellipsoid zone (EZ) and the external limiting membrane (ELM). Disruption was defined as any discontinuity or absence of the corresponding hyperreflective bands within the central 1-mm diameter zone on horizontal B-scans through the fovea. Two independent blinded graders evaluated the outer retinal integrity at all three time points.

### Visual acuity assessment

BCVA was measured using a standard Landolt C-chart at 5 meters under standardized lighting conditions. Visual acuity values were converted to the logarithm of the minimum angle of resolution (logMAR) for statistical analysis. All measurements were conducted by certified ophthalmic technicians.

### Data collection

The following data were extracted from medical records: patient demographics (age and sex); previous treatment history, including number and type of anti-VEGF injections, focal laser photocoagulation, and intravitreal or sub-Tenon corticosteroid injections; OCT parameters at three time points (1 year before switching, immediately before switching, and 1 year after switching); BCVA at the same three time points; number of faricimab injections administered during the 12-month post-switching period; and number of aflibercept or ranibizumab injections administered during the 12-month pre-switching period.

### Statistical analysis

The patient information required for statistical analysis is provided as S1 Table. Statistical analyses were performed using JMP software version 17 (SAS Inc., Cary, NC, USA). Continuous variables were expressed as mean ± standard deviation (SD) for normally distributed data or median with interquartile range (IQR) for non-normally distributed data. Normality was evaluated using the Shapiro–Wilk test. Categorical variables were expressed as frequencies and percentages.

To evaluate changes in CRT across three time points, a linear mixed model was employed to account for the correlation between eyes of the same patient, followed by Tukey’s honest significant difference (HSD) test for post-hoc pairwise comparisons. The same approach was used for CCT and visual acuity. Injection frequency before and after switching was compared using the paired Student’s t‑test.

The disruption of the ellipsoid zone (EZ) and external limiting membrane (ELM) was analyzed using a generalized linear mixed model (GLMM).

Subgroup analyses comparing responders (achieving CRT ≤ 325 μm at 1 year) and non-responders were performed using Student’s t-test or the Mann–Whitney U test, as appropriate.

Statistical significance was set at P < 0.05. No adjustment for multiple comparisons was applied given the exploratory nature of the CCT endpoint.

## Results

A total of 28 eyes from 19 patients met the inclusion criteria and were analyzed. Patient demographics and baseline characteristics are summarized in Table 1. The mean age was 66.6 ± 9.8 years, with 13 male (65.0%) and seven female patients (35.0%).

**Table 1:** Patient demographics and baseline characteristics.

Characteristic	Value
Demographics	
Number of patients	19
Number of eyes	28
Age (years), mean ± SD	66.0 ± 9.8
Sex, n (%)	
- Male	13 (65.0%)
- Female	7 (35.0%)
Previous treatment history	
Previous anti-VEGF agent, n (%)	
- Aflibercept only	26 (92.9%)
- Ranibizumab and aflibercept	2 (7.1%)
Previous focal laser, n (%)	7 (25.0%)
Previous sub-Tenon steroid, n (%)	1 (3.6%)
Baseline OCT characteristics	
CRT at 1 year before switching (µm), mean ± SD	398 ± 118
CRT immediately before switching (µm), mean ± SD	453 ± 112
CRT at 1 year after switching (µm), mean ± SD	322 ± 60
CCT at 1 year before switching (µm), mean ± SD	246 ± 76
CCT immediately before switching (µm), mean ± SD	238 ± 82
CCT at 1 year after switching (µm), mean ± SD	230 ± 62
EZ/ELM disruption at 1 year before switching, n (%)	8 (28.6%)
EZ/ELM disruption immediately before switching, n (%)	10 (35.7%)
EZ/ELM disruption at 1 year after switching, n (%)	11(39.3%)
Baseline visual acuity	
BCVA at 1 year before switching (logMAR), mean ± SD	0.31 ± 0.27
BCVA immediately before switching (logMAR), mean ± SD	0.31 ± 0.27
BCVA at 1 year after switching (logMAR), mean ± SD	0.34 ± 0.32

SD, standard deviation; IQR, interquartile range; VEGF, vascular endothelial growth factor; OCT, optical coherence tomography; CRT, central retinal thickness; CCT, central choroidal thickness; EZ, ellipsoid zone; ELM, external limiting membrane; BCVA, best-corrected visual acuity; logMAR, logarithm of the minimum angle of resolution.

Before switching to faricimab, 26 eyes (92.9%) had been treated exclusively with aflibercept, whereas two eyes (7.1%) had received both ranibizumab and aflibercept. The median number of anti-VEGF injections administered before enrollment was 5.5 (IQR: 4–9). Seven eyes (25.0%) had previously undergone focal laser photocoagulation, and one eye (3.6%) had received a sub-Tenon triamcinolone acetonide injection more than 6 months before switching.

**Fig 1:**
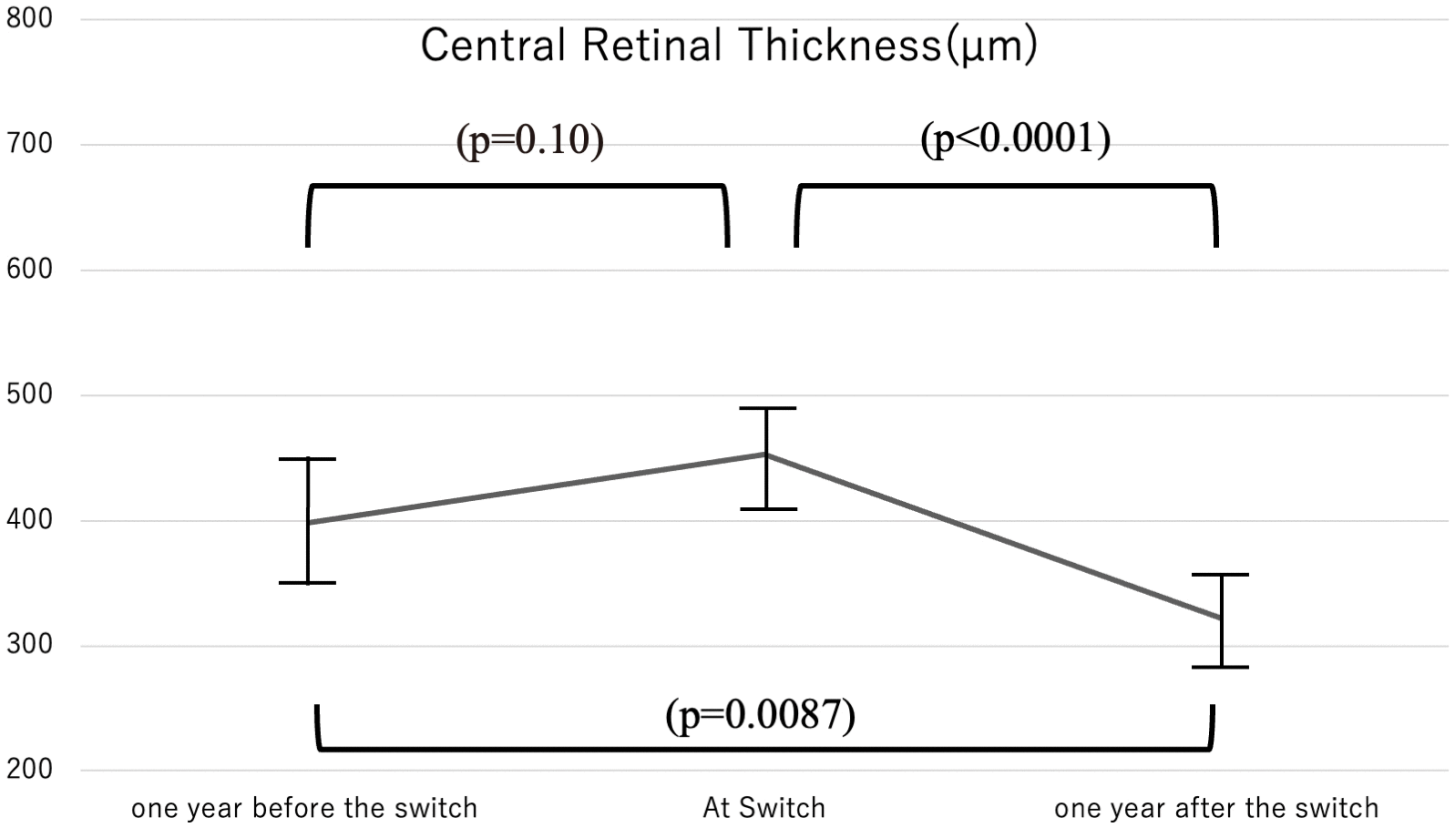
Average central retinal thickness (CRT) 1 year before switching, immediately before switching, and 1 year post-switching.

CRT demonstrated significant variations across the three study time points (Fig 1). Mean CRT increased from 398 ± 118 μm at 1 year before switching to 453 ± 112 μm immediately before switching. After switching to faricimab, CRT decreased to 322 ± 60 μm at 1 year after switching. CRT at 1 year post-switching decreased more significantly than immediately before and at 1 year before switching (P < 0.05, linear mixed model followed by Tukey’s HSD test).

The line diagram shows the mean CRT at three time points: 1 year before switching, immediately before switching, and 1 year post-switching. The mean CRT was 398 ± 118 µm 1 year before switching, 453 ± 112 µm immediately before switching, and 322 ± 60 µm 1 year post-switching. CRT measured 1 year post-switching showed a more significant decrease than that observed immediately before and 1 year before switching (P < 0.05, linear mixed model followed by Tukey’s honest significant difference [HSD] test).

The mean CRT reduction from immediately pre-switching to 1 year post-switching was 131.2 ± 137.7 μm, corresponding to a 28.9% decrease. At 1-year post-switching, 15 eyes (53.6%) achieved the dry macula threshold (CRT ≤ 325 μm).

**Fig 2:**
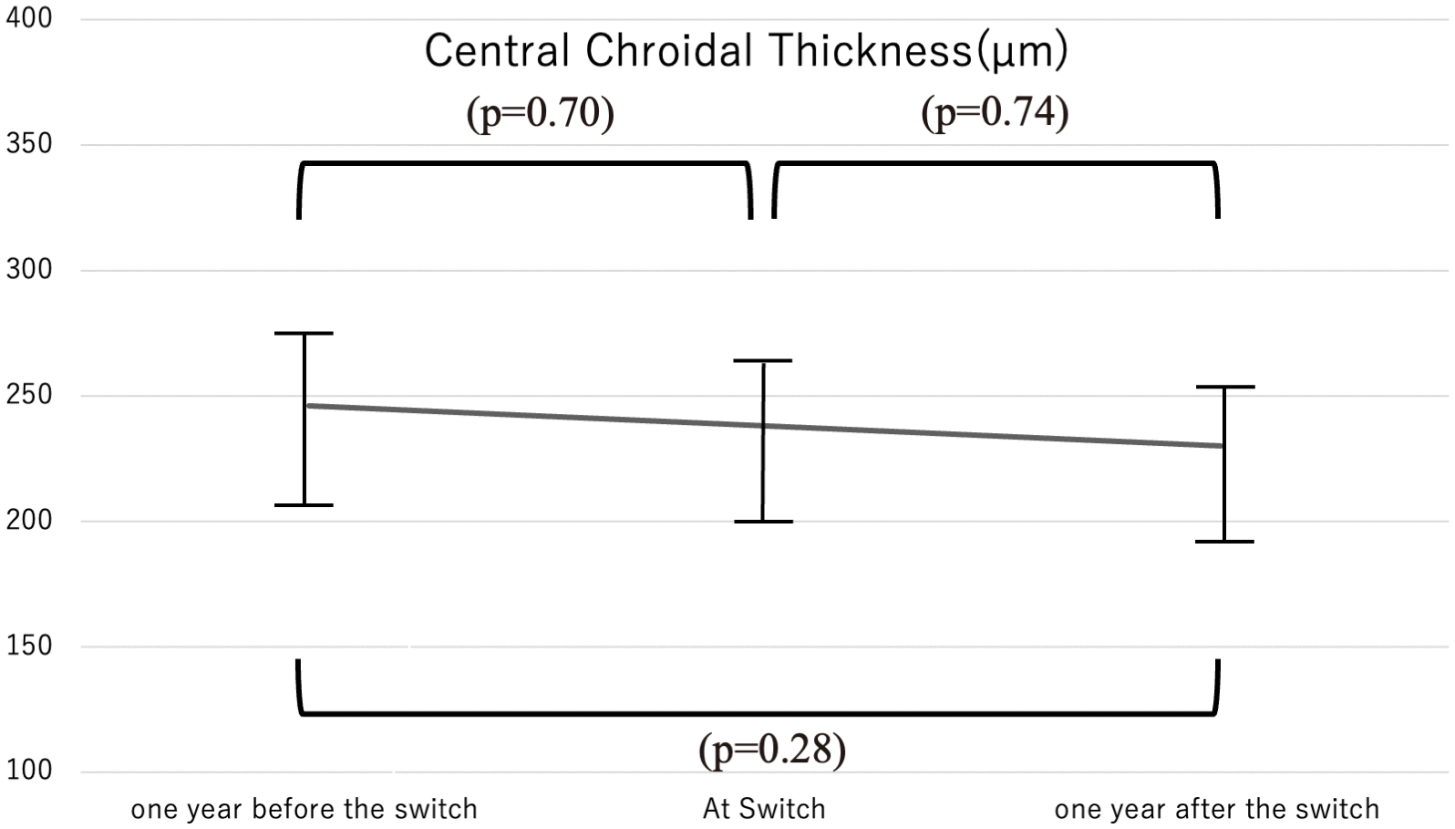
Average central choroidal thickness (CCT) at 1 year before switching, immediately before switching, and 1year post-switching.

As an exploratory outcome, changes in CCT were evaluated across the same time points (Fig 2). The mean CCT was 246 ± 76 μm at 1 year before switching, 238 ± 82 μm immediately before switching, and 230 ± 62 μm at 1 year post-switching. Linear mixed model revealed no significant changes across time points (P = 0.2288).

The line diagram shows the mean CCT at three time points: 1 year before switching, immediately before switching, and 1 year after switching. Mean CCT was 246 ± 76 μm at 1 year before switching, 238 ± 82 μm immediately before switching, and 230 ± 62 μm at 1 year after switching. Linear mixed model revealed no significant changes across time points (P = 0.2288).

Subgroup analysis comparing responders (n = 15) and non-responders (n = 13) showed that responders had a mean CCT change of −19.07 ± 13.08 μm, whereas non-responders had a change of 5.92 ± 14.05 μm (P = 0.204). Neither group demonstrated significant CCT alterations despite divergent retinal responses.

Outer retinal integrity also showed temporal trends. At 1 year before switching, eight eyes (28.6%) demonstrated EZ or ELM disruptions; this increased to 10 eyes (35.7%) immediately before switching (P > 0.05, linear mixed model [GLMM]). At 1 year post-switching, outer retinal disruption remained stable in 11 eyes (39.3%), representing only a 3.6% increase over the post-switching year.

**Fig 3:**
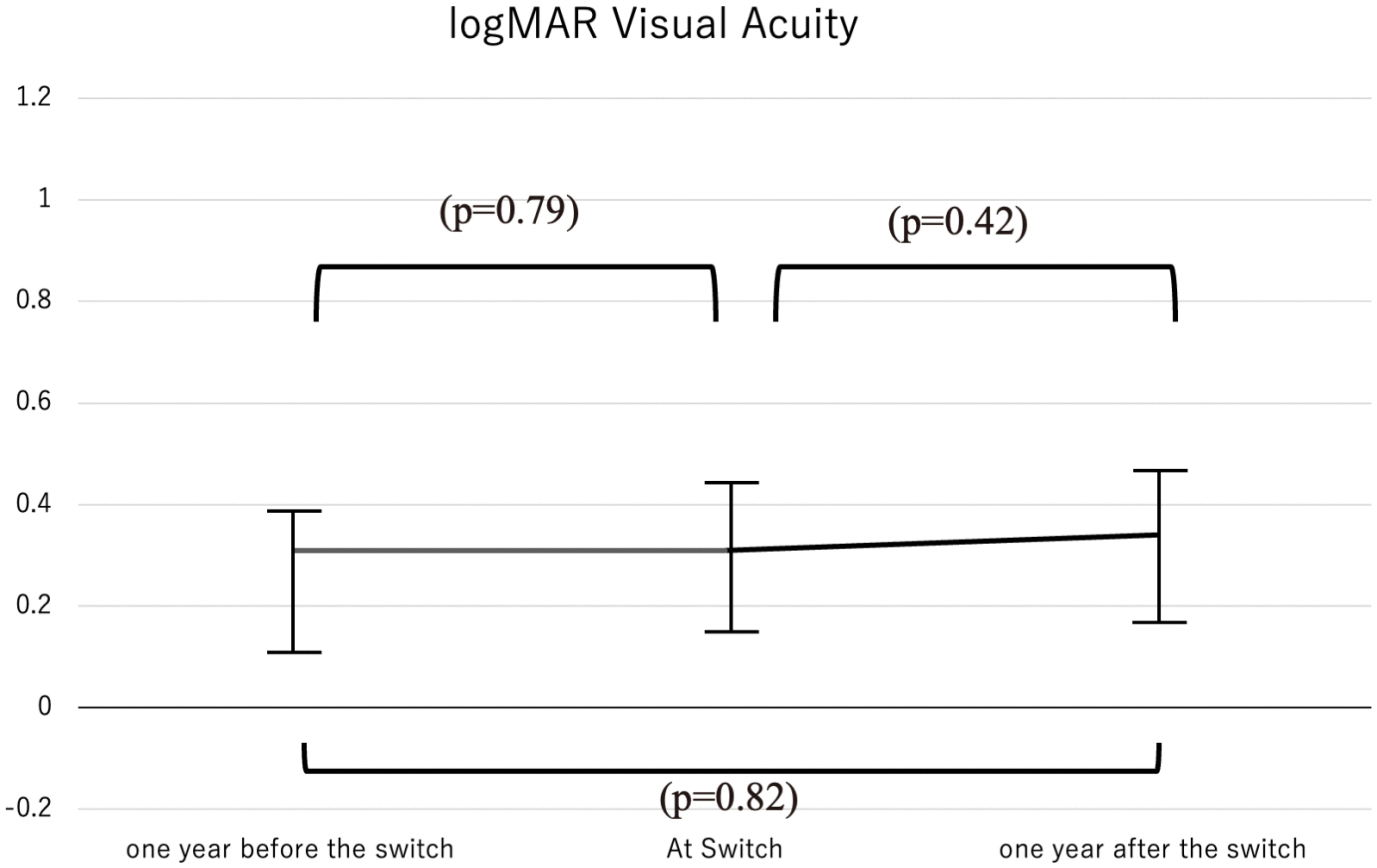
Average logarithm of the minimum angle of resolution (logMAR) best‑corrected visual acuity (BCVA) at 1 year before switching, immediately before switching, and 1 year after switching.

BCVA demonstrated no significant changes across all time points (Fig 3). LogMAR visual acuity was 0.31 ± 0.27 at 1 year before switching, 0.31 ± 0.27 immediately before switching, and 0.34 ± 0.32 at 1 year post-switching (P = 0.2740 by linear mixed model). Among the 15 eyes that achieved dry macula status at 1 year, the mean visual acuity demonstrated slight improvement from 0.296 ± 0.06 to 0.325 ± 0.06 logMAR, but this change did not reach statistical significance (P = 0.7519 by Student’s t-test).

Line diagram showing the mean logMAR BCVA at three time points: 1 year before switching, immediately before switching, and 1 year post-switching. LogMAR visual acuity was 0.31 ± 0.27 at 1 year before switching, 0.31 ± 0.27 immediately before switching, and 0.34 ± 0.32 at 1 year after switching (P = 0.2740 by linear mixed model).

**Fig 4:**
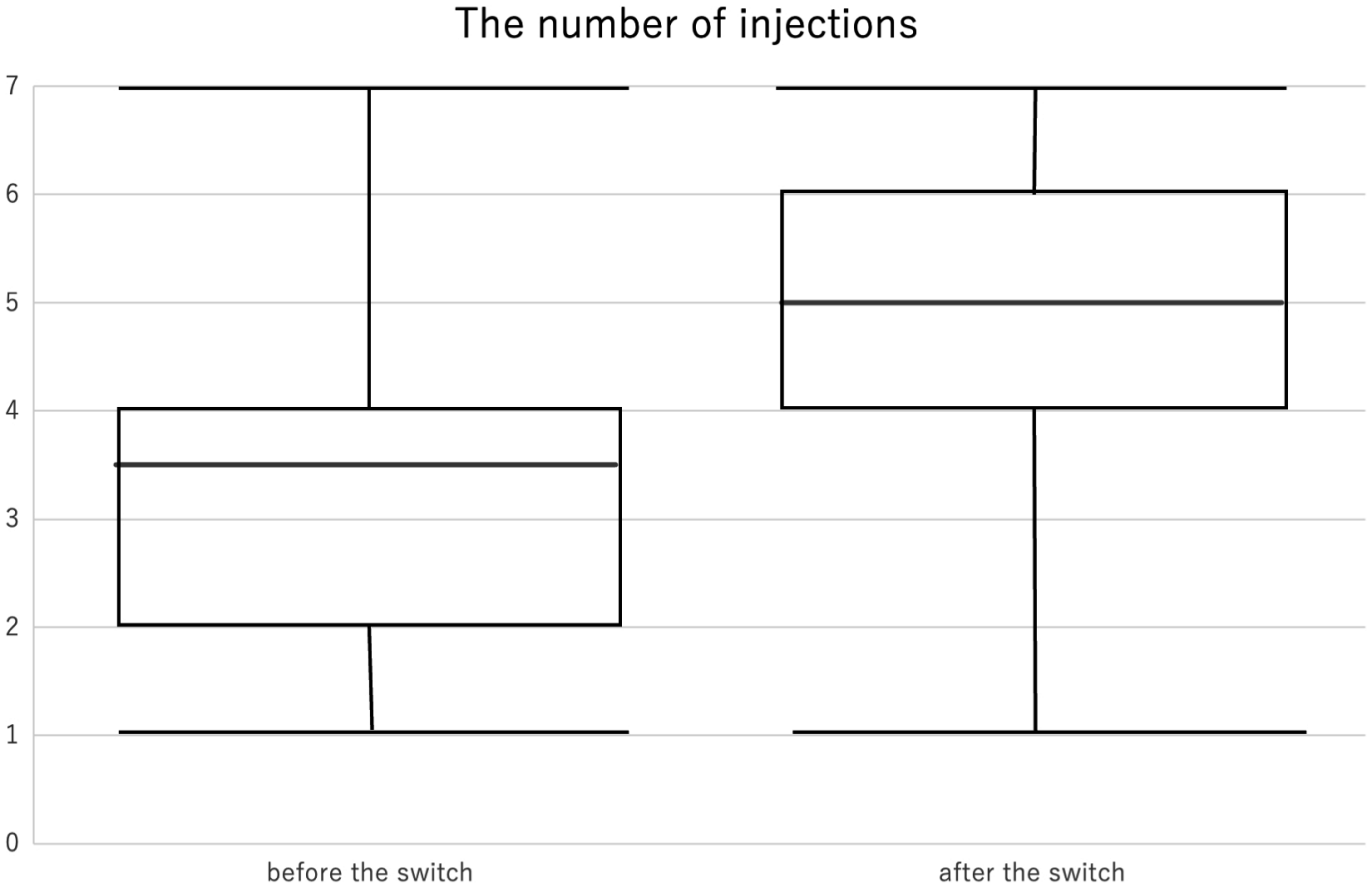
Number of injections in the 1-year period before and after switching.

The median number of intravitreal injections administered during the 1-year period before switching was 3.5 (2–4), reflecting the PRN regimen used with aflibercept or ranibizumab in these treatment-resistant cases. During the 1-year period post-switching under the 1 + PRN protocol, the mean number of injections was five (4–6) (Fig 4). The number of intravitreal injections was significantly higher during the 1-year period post-switching than that during the pre-switching period (Wilcoxon signed-rank test, P = 0.0003).

Box plots showing the number of intravitreal injections administered in the year before switching to faricimab (aflibercept or ranibizumab) and the year after switching to faricimab using the 1 + PRN protocol. The median number of injections in the 1 year before switching was 3.5 (2–4 injections), whereas after switching to faricimab it was five (4–6 injections). In the 1 year after switching to faricimab, the number of intravitreal injections was significantly higher than that in the pre-switching period (Wilcoxon signed-rank test, P = 0.0003).

**Fig 5:**
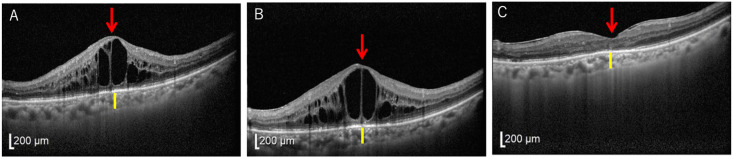
Representative optical coherence tomography (OCT) Images illustrating changes in central retinal thickness (CRT) and stability of central choroidal thickness (CCT) and external limiting membrane/ellipsoid zone (ELM/EZ) integrity over three time points.

Representative optical coherence tomography images illustrating the structural changes before and after switching to faricimab therapy are provided in Fig 5.

Images showing horizontal OCT B-scans through the fovea from one representative patient at three time points: 1 year before switching (Image A), immediately before switching (Image B), and 1 year post-switching (Image C). In Image A (1 year before switching), the CRT was 670 μm, and the CCT was 284 μm. Disruption of the ELM and EZ was already present at this time point. In Image B (immediately before switching), CRT increased to 740 μm and CCT increased to 291 μm, with persistent ELM/EZ disruption. In Image C (1 year post-switching), CRT improved to 244 μm with resolution of intraretinal cystic spaces, while CCT remained stable at 230 μm. The pre-existing ELM/EZ disruption demonstrated no further deterioration and was maintained without progression. Yellow calipers indicate subfoveal choroidal thickness measurement points, and red arrows indicate the locations of choroidal measurements. Scale bar = 200 μm.

## Discussion

This real-world observational study demonstrated that switching to faricimab in treatment-resistant DME resulted in significant anatomical improvement, with a 29% reduction in mean CRT and dry macula achievement in 53.6% of eyes at 1 year. These findings support the efficacy of faricimab switching therapy in refractory cases and align with recent reports from other real-world cohorts [13–16]. Notably, CCT remained stable during faricimab therapy despite a substantial reduction in retinal thickness, with no apparent relationship between the retinal and choroidal responses.

Our primary finding of significant CRT reduction after switching to faricimab confirms and extends previous real-world observations. Pichi et al. reported that poor responders to aflibercept demonstrated a meaningful BCVA increase (+ 5 letters, P = 0.007) and CRT reduction (−67.9 μm, P = 0.004) after switching to faricimab [13]. Huber et al. demonstrated that 54% of aflibercept responders and 25% of non-responders achieved significant anatomical improvement at 12 weeks post-switching [14]. Tatsumi et al. noted that multiple faricimab injections (≥ 3) were required for optimal outcomes, with a significant CRT reduction by the third injection [15]. Rush reported that 82.4% of patients with ≥ 50 μm CRT reduction after the first three faricimab injections achieved ≥ 8-week treatment intervals with a fluid-free macula at 12 months [16]. Our results align with these reports, with the added value of a longer follow-up and the inclusion of CCT measurements. The magnitude of anatomical improvement observed in our study is notable, given the refractory nature of the included cases. All patients exhibited persistent edema with CRT ≥ 325 μm despite regular anti-VEGF therapy with aflibercept or ranibizumab. The marked reduction in CRT and high dry macula rate achieved following faricimab supports the concept that dual inhibition of VEGF-A and Ang-2 offers additional therapeutic benefits when VEGF-A blockade alone is insufficient [10,11].

The enhanced efficacy observed with faricimab is likely attributable to the role of Ang-2 in DR pathogenesis. Ang-2 promotes vascular instability, inflammation, and permeability through antagonism of Tie2 receptor signaling [8,9]. Elevated Ang-2 levels in the aqueous humor have been linked to poor response to anti-VEGF monotherapy [10], indicating that patients with high Ang-2 expression may benefit from dual inhibition. Future studies incorporating biomarker analysis may help identify individuals who are most responsive to faricimab after switching.

While the high central retinal thickness (CRT) observed immediately prior to the switch might raise concerns regarding potential selection bias by the examining physicians—specifically, the possibility that the switch was considered only when edema worsened—we believe this was likely mitigated by our study design. The transition to faricimab was performed for all eyes that met the predefined criteria for treatment-resistant diabetic macular edema (DME) during clinical visits following May 2022, when the agent was first approved for DME in Japan. Consequently, the selection of cases for the switch was determined based solely on this timeframe, regardless of the severity of the macular edema or the degree of prior treatment control at that point. Furthermore, as the study population was limited to patients managed by the lead author and co-authors, we consider it less likely that subjective clinical judgments regarding treatment changes by other physicians influenced the results. On the other hand, the increase in CRT immediately prior to the switch may partially reflect a so-called regression to the mean, which may have made the changes observed one year after the switch appear more significant.

Despite substantial anatomical improvement, no significant visual acuity gain was observed after switching to faricimab. This mismatch between anatomical and functional outcomes has been consistently reported in switch studies of treatment-resistant DME [13–16] and likely reflects irreversible outer retinal damage from chronic edema.

Outer retinal disruption was present in 28.6% of eyes at 1 year before switching and 35.7% immediately before switching, indicating progression during the pre-switching period despite ongoing DME treatment. However, at 1 year after switching, outer retinal disruption increased only slightly to 39.3%, a modest increase of 3.6%. This limited progression is notable because chronic DME typically leads to progressive photoreceptor damage and outer retinal atrophy [27,28]. Despite the chronic nature of DME in our cohort, the minimal progression of outer retinal disruption during the post-switching period indicates that faricimab therapy may suppress further structural deterioration.

The EZ represents the inner segment/outer segment junction of the photoreceptors and is essential for visual function. Disruption of this layer is strongly related to poor visual outcomes in DME irrespective of anatomical improvements [3]. Maintaining outer retinal integrity is critical for visual recovery in patients with DME [27]. Zur et al. found that an intact EZ at baseline was the strongest predictor of visual acuity improvement following anti-VEGF therapy [29]. In this context, outer retinal deterioration during faricimab therapy may reflect a protective effect that helps preserve remaining visual function and prevent further decline.

These findings highlight the importance of initiating DME treatment early, before irreversible structural damage. They also emphasize the need for realistic expectations when switching therapies in patients with long-standing refractory diseases. Although anatomical improvements may reduce the risk of further vision loss and potentially enhance retinal oxygenation and metabolism, functional recovery remains limited by preexisting damage. Nevertheless, the observed suppression of outer retinal deterioration suggests that faricimab may provide benefits beyond simple edema reduction, possibly through enhanced vascular stability and reduced chronic inflammation [30,31].

The median injection frequency in our study significantly increased following switching to faricimab (3.5 [2–4] to five [4–6] injections per year). This finding differs from those of the YOSEMITE and RHINE trials, which reported extended dosing intervals in treatment-naïve patients [12]. However, our cohort consisted exclusively of treatment-resistant patients with chronic edema, representing a fundamentally different patient group.

In refractory cases, in principle, follow-up examinations were scheduled monthly throughout the study period though, for certain patients receiving very long-term aflibercept treatment, follow-up intervals had been clinically extended to every few months prior to the switch. We believe this was due to the refractory nature of their macular edema, which had reached a “treatment plateau” where further anatomical improvement was minimal despite prolonged therapy. In such instances, it was clinically judged that additional injections of the same agent would yield limited further benefits. Conversely, following the switch to faricimab, monthly follow-ups were strictly implemented for all cases to accurately evaluate the efficacy of the new agent and to optimize the treatment protocol. This difference in the management phase may have resulted in a relatively shorter average follow-up interval after the switch compared to the pre-switch period. Consequently, the injection frequency in the post-switch period might appear higher than it was previously, a factor that should be considered when interpreting the results. However, the observed anatomical improvements, despite the long-term refractory nature of the edema, suggest that the switch to faricimab provided a valuable clinical opportunity to reconstruct the treatment approach and move beyond the previous therapeutic limits.

Importantly, the primary objective of switching therapy in refractory cases is to achieve disease control rather than to reduce the injection burden. Therefore, maintaining a similar injection frequency while achieving superior anatomical outcomes represents a successful therapeutic strategy. Future studies examining more structured loading regimens or combination therapies may help optimize dosing for this challenging population.

The stability of CCT, despite substantial retinal thickness reduction, is a novel finding that has not been previously reported with faricimab. While macular edema and increased retinal thickness are common features of DME, the behavior of CCT remains controversial. Some studies have reported that CCT remained unchanged in patients with DME when compared with healthy controls [18], while others have found variable alterations depending on disease severity [19–21] and treatment [22–24].

Previous studies on anti-VEGF effects on CCT in DME have demonstrated variable results depending on the agent used [22–24]. Notably, some investigators have suggested that choroidal thinning during anti-VEGF therapy may reflect impaired vascular perfusion rather than resolution of choroidal congestion, raising concerns about the potential adverse effects on choroidal circulation [25].

Our observation of CCT stability is particularly notable in light of recent functional data from Saima et al., who demonstrated reduced ocular blood flow in the optic disc region after switching from aflibercept to faricimab [26]. Although their analysis focused on retinal blood flow, when combined with our anatomical findings, these findings suggest that faricimab may effectively reduce retinal oedema by decreasing retinal blood flow, whilst preserving the choroidal vasculature.

The Ang-2–Tie2 pathway plays a critical role in choroidal vascular homeostasis and endothelial stability [32,33]. Balanced inhibition of both VEGF-A and Ang-2 may preserve choroidal perfusion while effectively treating retinal edema, potentially by maintaining pericyte coverage and endothelial junction integrity in the fenestrated choroidal vasculature [32,33].

Although these central choroidal ‌‌thickness (CCT) findings remain exploratory, the fact that the reduction in choroidal thickness following the switch was kept to a minimum suggests that it may serve as a potential marker for the maintenance of vascular health. Choroidal thinning has been linked to outer retinal atrophy and poor visual outcomes in some studies [18], indicating that the preservation of choroidal structure and function may be beneficial. It is widely recognized that choroidal circulation supplies a substantial proportion of the nutrients to the outer retina. In addition, previous studies have reported that vascular endothelial growth factor (VEGF) secreted by the retinal pigment epithelium plays an essential role in maintaining the choriocapillaris [34]. Considering these physiological mechanisms, it cannot be completely ruled out that excessive suppression of VEGF may be associated with reduced choroidal blood flow and, consequently, impaired circulation in the outer retina.

From this perspective, our finding that central choroidal thickness (CCT) did not show a marked decrease after switching may also suggest that faricimab contributes to the maintenance of choroidal circulation. However, we acknowledge that CCT has not been established as a standard treatment indicator for diabetic macular edema. Therefore, the relationship between central retinal thickness (CRT) and CCT observed in the present study should be regarded as exploratory. Further research with larger sample sizes, a prospective design, and functional choroidal evaluations (such as choroidal blood flow measurements or OCT angiography) is required to confirm these preliminary observations and establish their clinical significance.

This study has some limitations. First, the retrospective observational design without a control group restricts causal inferences. Although natural fluctuations or regression to the mean cannot be entirely excluded, the magnitude and consistency of CRT reduction make these explanations less likely. Second, the sample size was relatively small (28 eyes), which limited the statistical power of the subgroup analyses and the ability to detect modest effects. Third, the observation that CRT (central retinal thickness) had increased immediately prior to the switch may partially reflect a “regression to the mean.” Consequently, the changes observed one year after the switch might have appeared more pronounced than they were. Fourth, the CCT measurements were conducted manually rather than by automated segmentation, introducing potential measurement variability. However, the use of two independent masked graders minimized this limitation. Fifth, only anatomical CCT was assessed, without complementary functional evaluations such as choroidal blood flow or vascular density, using OCT angiography. Such functional assessments would provide stronger insights into the interpretation of the CCT findings. Sixth, the 1-year follow-up period, while sufficient to evaluate initial treatment response, may not capture long-term outcomes or late treatment effects. Finally, our cohort was treated at a single academic center in Japan, which may restrict the generalizability to other populations or healthcare settings. The predominantly Asian patient population may have different choroidal characteristics than those of other ethnic groups, as CCT has been shown to vary with ethnicity [19]. Multicenter studies with diverse populations would improve the external validity of our findings.

Despite these limitations, this study has several important clinical implications. First, it provided real-world validation supporting faricimab as an effective switch therapy for treatment-resistant DME, achieving anatomical improvement in most cases. Second, it established realistic expectations regarding visual outcomes in refractory cases where structural damage limited functional recovery. Third, the minimal progression of outer retinal disruption suggests that faricimab may provide protective effects beyond simple edema reduction. Fourth, the novel observation of stable CCT during therapy raises the possibility that choroidal thickness may serve as an indicator of vascular health.

In conclusion, switching to faricimab in treatment-resistant DME produces significant anatomical improvement with a 28.9% reduction in CRT, validating its efficacy in real-world refractory cases. Visual acuity improvement is restricted by pre-existing outer retinal damage; however, the minimal progression of outer retinal disruption during faricimab therapy compared with the pre-switching period suggests potential suppression of structural deterioration, helping preserve residual visual function. Although the burden of treatment has not been reduced compared to before the switch, indicating that the primary benefit was disease control rather than reduced injection frequency. CCT stability may reflect preserved choroidal perfusion with dual VEGF-A and Ang-2 inhibition. This observation warrants further study for a potential marker of a favorable vascular response. Overall, these findings support faricimab as an effective switch therapy option for treatment-resistant DME and highlight outer retinal preservation and choroidal assessment as valuable areas for future research.

## Supporting information

S1 Table Baseline characteristics of the study population. Clinical characteristics of patients before and after switching at 1 year before switching, immediately before switching, and 1 year after switching.
(PDF)
