# Supplementary material for: Real-world outcomes after switching to faricimab in treatment-resistant diabetic macular edema: A 1-year observational study with choroidal thickness assessment
Source: PLoS One. 2026 Jun 10;21(6):e0351168. doi: 10.1371/journal.pone.0351168 (PMC13252804; doi:10.1371/journal.pone.0351168)
Supplement: S1 Table — Clinical characteristics of patients before and after switching at 1 year before switching, immediately before switching, and 1 year after switching. (PDF) [file pone.0351168.s001.pdf]

case(No)	patient(No)	1 year before switching				immediately before-switching				1-year post-switching			
		logMAR BCV	CRT (μm)	CCT (μm)	the presence of EZ/ELM disruption	logMAR BCV	CRT (μm)	CCT (μm)	the presence of EZ/ELM d	logMAR	CRT	CCT	the presence of EZ/ELM disruption
casae1	1	0.69897	259	284	+	0.69897	740	291	+	0.82390874	244	230	+
casae2	1	0.52287875	579	289	—	0.30103	708	218	+	0.39794001	245	152	+
casae3	2	0.04575749	425	134	—	0.15490196	361	101	—	0.09691001	305	247	—
casae4	3	0	419	143	—	-0.0791812	466	101	—	-0.0791812	437	198	—
casae5	4	0.09691001	724	274	—	0.30103	624	268	—	0.09691001	291	218	—
casae6	5	0.69897	411	235	+	0.82390874	436	182	+	1	327	175	+
casae7	6	0.30103	341	265	—	0.22184875	486	258	—	0.15490196	315	265	—
casae8	7	0.52287875	494	150	+	0.69897	527	149	+	0.52287875	323	154	+
casae9	8	0.04575749	487	228	—	0.22184875	489	229	—	0.30103	377	219	—
casae10	9	0.09691001	356	272	—	0.09691001	347	322	—	0.09691001	302	303	—
casae11	10	0	258	124	+	0.30103	486	124	+	0.22184875	233	131	+
casae12	10	0.39794001	337	114	+	0.30103	344	127	+	0.30103	246	122	+
casae13	11	0.30103	215	235	—	0.39794001	199	253	+	0.82390874	372	238	+
casae14	11	0.09691001	251	292	—	0.09691001	440	320	—	0.09691001	283	305	—
casae15	12	0.30103	338	365	—	0.30103	371	383	—	0.30103	328	308	+
casae16	12	0.69897	396	375	—	0.52287875	493	389	—	0.52287875	309	338	—
casae17	13	-0.0791812	371	255	—	0	396	238	—	0.09691001	279	228	—
casae18	14	-0.0791812	366	229	+	0.04575749	504	237	+	0.22184875	328	232	+
casae19	15	0.22184875	437	342	—	0.22184875	403	356	—	0.82390874	258	221	—
casae20	15	0.52287875	537	365	—	0.30103	470	278	—	0.69897	256	252	—
casae21	16	0.09691001	397	130	—	0.22184875	351	125	—	0.04575749	314	110	—
casae22	16	0.30103	395	229	—	0.30103	396	185	—	0.09691001	287	194	—
casae23	17	0.30103	644	268	+	0.22184875	521	265	+	0.30103	420	283	+
casae24	17	-0.0791812	290	218	—	0	391	191	—	-0.0791812	331	233	—
casae25	18	0	315	355	—	0.04575749	472	336	—	0.22184875	419	343	—
casae26	18	0.22184875	410	275	—	0.22184875	523	285	—	0.15490196	402	305	—
casae27	19	1.04575749	384	242	+	1.09691001	374	235	+	1	377	241	+
casae28	19	0.52287875	310	205	—	0.69897	374	223	—	0.30103	411	215	—
