# Supplementary material for: Real-world outcomes after switching to faricimab in treatment-resistant diabetic macular edema: A 1-year observational study with choroidal thickness assessment
Source: PLoS One. 2026 Jun 10;21(6):e0351168. doi: 10.1371/journal.pone.0351168 (PMC13252804; doi:10.1371/journal.pone.0351168)
Supplement: Attachment — Submitted filename: Response to Reviewers.docx [file pone.0351168.s002.docx]

Dear Daisuke Nagasato, Academic Editor,

We wish to express our appreciation to the editors and reviewers for their insightful comments, which have helped us significantly improve the paper. We have revised the manuscript (PONE-D-26-10259) according to the reviewer s’ comment.

We hope that our manuscript is now suitable for publication in PLOS One.

.

Sincerely,

Hidetsugu Mori, MD+PhD
Ryo Tomemori, MD

Real-world outcomes after switching to faricimab in treatment-resistant diabetic macular edema: A 1-year observational study with choroidal thickness assessment

PONE-D-26-10259

Reply to Reviewer #1: Overall assessment

This is a valuable real-world report evaluating the clinical significance of a newer anti-VEGF agent for diabetic macular edema (DME), and the inclusion of choroidal thickness assessment is particularly interesting. If the key methodological and interpretive concerns below can be addressed through additional data/analyses and clearer descriptions, the manuscript could become a highly impactful contribution to the field.

Major comments

1) Data availability / transparency

The Data Availability statement indicates that “All relevant data are within the manuscript and its Supporting Information files.” However, as currently provided, I could not locate case-level/underlying data (e.g., per-eye CRT/CCT/BCVA values at each time point, injection dates/counts, or the dataset used for the analyses) in either the main text or the Supporting Information. It is possible that the relevant Supporting Information file(s) were not uploaded or were omitted during submission.

For transparency and reproducibility, please either (i) provide the underlying dataset as Supporting Information (de-identified, at least at the eye level), or (ii) revise the Data Availability statement to accurately reflect what is available and how the data can be accessed (e.g., upon reasonable request, subject to IRB restrictions), in accordance with journal policy.

We thank the reviewer for this comment. As you indicated, we have uploaded the case information as a Supporting Information file (ⅰ). We have also added the following to the ‘Statistical analysis’ section:(page10, line169-170)

2) Potential confounding by increased injection frequency (treatment burden not clearly “comparable”)

A central concern is that, although anatomical outcomes (e.g., CRT) improved after switching from aflibercept to faricimab, the injection frequency increased in the post-switch period. Therefore, it remains difficult to exclude the possibility that similar anatomical improvement might have been achieved simply by increasing injection frequency with aflibercept (i.e., an intensification effect rather than a switch effect). In this context, the Abstract statement “with a comparable treatment burden” appears overstated and should be revised or more carefully qualified.

We thank the reviewer for this comment. As you indicated, we acknowledge that the phrase 'with a comparable treatment burden' was an inappropriate description that could overstate the efficacy of faricimab, especially given that the injection frequency increased in the post-switch period. Accordingly, we have removed ‘with a comparable treatment burden’ from the manuscript in the abstract (page3, line 35–36.)

3) Visit schedule under the “1+PRN” regimen and its relationship to injection frequency)

The manuscript states that faricimab was administered using a “1+PRN” regimen, but it is unclear how often patients were actually reviewed (e.g., monthly vs. longer intervals). In real-world practice, follow-up intervals can vary substantially across institutions and even within the same center, depending on patient preferences and logistics. Please clarify the visit schedule after switching and, if applicable, before switching.

Specifically, I recommend reporting:
- The distribution of follow-up intervals (e.g., median and IQR, or a histogram) in the pre-switch and post-switch periods; -
- Whether visit intervals changed after switching (and in what direction);
- The relationship between visit interval and injection frequency (e.g., correlation or another appropriate analysis).

If the follow-up interval shortened after switching, this could readily explain the increased injection frequency, and it would be important to account for this when interpreting the post-switch outcomes.

Thank you for your insightful comment. In principle, the follow-up interval was monthly both before and after the switch, as stated on line 129 of the Abstract. However, among patients who had received very long-term treatment with aflibercept, some cases included intervals that had been extended to once every few months based on clinical judgment. We believe this was because these patients had refractory macular edema with an insufficient response to conventional anti-VEGF agents, reaching a "treatment plateau" where further improvement was minimal despite prolonged therapy. In such cases, it was clinically judged that additional injections of the same agent were unlikely to yield further benefits.

On the other hand, after switching to faricimab, monthly follow-up was implemented for all cases to accurately evaluate the efficacy of the new agent and to optimize the treatment protocol. We consider that this difference in the management phase may have relatively shortened the average follow-up interval after the switch, potentially making the actual injection frequency appear higher than it was during the pre-switch period.

Regarding the suggested additional analysis, we found it difficult to perform a robust evaluation due to the high degree of variability in frequency among individual patients. However, we have explicitly stated these circumstances in the revised manuscript as described below to ensure the transparency of our data, and added the following text, replacing the original content to take the context into account. (page24, line386-406)

Several factors may account for the inability to extend the dosing intervals in refractory cases. First, these patients exhibited aggressive disease biology that showed resistance to conventional therapy, indicating a more severe underlying pathology. Second, chronic inflammation and structural vascular changes may necessitate more frequent interventions to maintain fluid control. Third, our use of a 1+PRN protocol rather than a treat-and-extend regimen may have resulted in different dosing patterns from those in clinical trials that employed more structured treatment algorithms.

↓

‘In principle, follow-up examinations were scheduled monthly throughout the study period. However, for certain patients receiving very long-term aflibercept treatment, follow-up intervals had been clinically extended to every few months prior to the switch. We believe this was due to the refractory nature of their macular edema, which had reached a "treatment plateau" where further anatomical improvement was minimal despite prolonged therapy. In such instances, it was clinically judged that additional injections of the same agent would yield limited further benefits.’

Conversely, following the switch to faricimab, monthly follow-ups were strictly implemented for all cases to accurately evaluate the efficacy of the new agent and to optimize the treatment protocol. This difference in the management phase may have resulted in a relatively shorter average follow-up interval after the switch compared to the pre-switch period. Consequently, the injection frequency in the post-switch period might appear higher than it was previously, a factor that should be considered when interpreting the results. `

4) Interpretation of “worsening despite ongoing therapy” and risk of reverse causality / selection bias

In the Results, the manuscript states:
“Mean CRT increased from 398 ± 118 μm at 1 year before switching to 453 ± 112 μm immediately before switching, indicating progressive edema despite ongoing anti-VEGF therapy.”

Two points here:

(a) Placement and tone: The interpretive phrase (“indicating progressive edema…”) is more appropriate for the Discussion than the Results section, and the conclusion should be stated cautiously.

We thank the reviewer for this comment. As suggested, the phrase 'indicating progressive edema...' was not appropriate for the Results section. Accordingly, we have removed this expression in the Abstract (page16, line 247).

(b) Reverse causality / selection bias: In practice, switching often occurs because disease worsened or was perceived to be insufficiently controlled, rather than the worsening being evidence of inefficacy under a stable treatment strategy. In addition, because faricimab became available during the study period, it is unlikely that all eligible patients were switched immediately upon meeting a predefined retreatment threshold; patients with good control may be less likely to request/accept switching, and clinicians may be more inclined to recommend switching after demonstrating persistent/worsening edema. Thus, the observed increase in mean CRT immediately before switching may reflect “patients were switched when they worsened,” rather than “patients worsened despite therapy,” i.e., the direction of causality may be reversed.

To address this, please provide a clear explanation of:
- The explicit criteria and workflow used to decide when to switch (beyond the CRT threshold);
- Whether switching was triggered at a single visit with transient worsening versus sustained inadequate response across multiple visits;
- Any steps taken to mitigate selection bias.

We thank the reviewer for this comment. The timing of switching therapy was restricted to a single visit after May 2022, when faricimab was approved for the treatment of DME in Japan. All eyes diagnosed with treatment-resistant macular edema according to the definition described in the abstract (page8,line 129) were switched to faricimab. Importantly, the selection of cases for switching was determined solely based on the period after May 2022, regardless of the severity of macular edema or the degree of treatment control. Therefore, we believe that this approach helped reduce potential selection bias.

In addition, this study included only patients managed by the first author and co-author (H.M) for research consistency, and therefore subjective treatment-switching decisions by other physicians were less likely to be reflected in the study population. This point has been clarified in the revised draft as follows.

Relatedly, the post-switch CRT reduction could partly reflect regression to the mean if switching tended to occur at a temporarily worse time point. Given the potential clinical impact of this type of real-world switch report on therapeutic adoption, careful framing in the Discussion (and an explicit statement in the Limitations) is warranted. While the data suggest potential benefit (including improvement compared with 1 year pre-switch), the possibility that part of the observed effect is explained by visit/injection intensity and/or regression to the mean should be transparently discussed.

We thank the reviewer for this comment. As suggested, considering that macular edema worsened from one year before the switch until immediately prior to the switch, we agree that this finding may partially reflect a regression to the mean.

Considering the two points you raised above, I have added the following text to the ‘Discussion’ section (page21-22, lines 337–350):

‘While the high central retinal thickness (CRT) observed immediately prior to the switch might raise concerns regarding potential selection bias by the examining physicians—specifically, the possibility that the switch was considered only when edema worsened—we believe this was likely mitigated by our study design. The transition to faricimab was performed for all eyes that met the predefined criteria for treatment-resistant diabetic macular edema (DME) during clinical visits following May 2022, when the agent was first approved for DME in Japan. Consequently, the selection of cases for the switch was determined based solely on this timeframe, regardless of the severity of the macular edema or the degree of prior treatment control at that point. Furthermore, as the study population was limited to patients managed by the lead author and co-authors, we consider it less likely that subjective clinical judgments regarding treatment changes by other physicians influenced the results.’

As suggested by the reviewer, we have added a discussion regarding "regression to the mean" in the Limitation section as follows (page27, line468-471)

`Third, the observation that CRT (central retinal thickness) had increased immediately prior to the switch may partially reflect a "regression to the mean." Consequently, the changes observed one year after the switch might have appeared more pronounced than they were. `

As a result of inserting ‘Third,’ the following changes have been made on page 28 of the abstract: ‘Third’ has been changed to ‘Fourth’ (line 471), ‘Fourth’ to ‘Fifth’ (line 474), and ‘Fifth’ to ‘Sixth’ (line 477).

5) Interpretation of CCT stability and outer retinal integrity changes

Several interpretive statements may also be influenced by the same confounding factors noted above.

- Regarding the sentence: “Notably, CCT remained stable during faricimab therapy despite a substantial reduction in retinal thickness, with no apparent relationship between the retinal and choroidal responses.” In real-world DME practice, switching decisions are typically driven by retinal findings (e.g., CRT/fluid), whereas CCT is not used clinically to guide switching. Therefore, the apparent dissociation between retinal and choroidal responses may be expected and does not necessarily support a mechanistic inference. Please consider tempering the causal/mechanistic interpretation and framing this as exploratory.

We thank the reviewer for this comment.

Given that the choroidal blood flow is responsible for the majority of the nutritional supply to the outer retinal layers, and considering that VEGF secreted from the retinal pigment epithelium plays a crucial role in maintaining the choroidal vasculature (○Reference: Saint-Geniez M, et al. Proc Natl Acad Sci U S A. 2009), we believe it is possible that excessive suppression of VEGF could lead to a reduction in choroidal blood flow and, consequently, be associated with circulatory disturbances in the outer retina. In this regard, since the decrease in CCT after switching was not marked in the present study, we would like to include the interpretation that faricimab maintains choroidal blood flow.

However, as correctly pointed out, CCT has not been established as a standard treatment guideline for diabetic macular edema to date. Therefore, we agree with the suggestion to remain cautious in describing the correlation between central retinal thickness (CRT) and CCT results, and to position this part of the study as exploratory. Furthermore, we believe that further long-term investigation regarding CCT is necessary, and we have added a statement to this effect in the abstract (page26-27, line446-452).

‘It is widely recognized that choroidal circulation supplies a substantial proportion of the nutrients to the outer retina. In addition, previous studies have reported that vascular endothelial growth factor (VEGF) secreted by the retinal pigment epithelium plays an essential role in maintaining the choriocapillaris (34). Considering these physiological mechanisms, it cannot be completely ruled out that excessive suppression of VEGF may be associated with reduced choroidal blood flow and, consequently, impaired circulation in the outer retina.

From this perspective, our finding that central choroidal thickness (CCT) did not show a marked decrease after switching may also suggest that faricimab contributes to the maintenance of choroidal circulation.

However, we acknowledge that CCT has not been established as a standard treatment indicator for diabetic macular edema. Therefore, the relationship between central retinal thickness (CRT) and CCT observed in the present study should be regarded as exploratory.

Further long-term investigations focusing on CCT will be necessary to validate these findings. `

From the perspective of conjunctions, I have removed ‘However,’ in the abstract (page27, line 458).

- Regarding outer retinal integrity: “Outer retinal disruption … increased from 28.6% to 35.7% … indicating progression … however … increased only slightly to 39.3% …” This pattern may also be at least partly explained by increased injection frequency and/or altered visit intervals after switching. Please consider addressing this possibility in the Discussion and/or Limitations and avoid attributing suppression of progression to the switch unless supported by additional analyses.

As you pointed out, these phrases were not appropriate for the ‘Results’ section. I have deleted the phrase ‘indicating progression during the pre-switching period’ （page16, line 247-8）, and the phrase ‘suggesting that faricimab therapy may inhibit outer retinal structural deterioration typically observed in chronic DME [27,28]’ (page16, line250-251)

We have also made the following changes to the main text to ensure that the results are not interpreted as attributing the suppression of disease progression to the change in treatment (page26, line439-443).

`While exploratory, these CCT findings raise the possibility that stable choroidal thickness during anti‑VEGF therapy could serve as a marker of preserved vascular health. `

↓

`Although these central choroidal thickness (CCT) findings remain exploratory, the fact that the reduction in choroidal thickness following the switch was kept to a minimum suggests that it may serve as a potential marker for the maintenance of vascular health. `

Overall, if the authors can address the above issues—particularly by clarifying switching criteria, visit intervals, and the relationship between injection frequency and outcomes—many of these interpretive concerns could be substantially alleviated, and the manuscript would become a very important and clinically useful contribution.


Minor comment

The statement that real-world evidence “remains limited” is supported by the subsequent paragraph summarizing several switch studies; however, for readability, it may be helpful to add the key citations directly after this sentence (e.g., refs. 13–16).

We thank the reviewer for this comment. As suggested. I have added [13–16] at the end of line 66 (page5).

Reply to Reviewer #2:

 The authors retrospectively investigated the visual outcome and OCT parameters before and after switching the anti-VEGF agents to faricimab in patients with refractory diabetic macular edema. This is an important theme in common practice. However, there are several issues that should be addressed.

I have a concern about statistical analyses. The authors included 28 eyes of 20 patients. Repeated measures ANOVA in its classical form cannot handle a mixed-eye dataset containing both unilateral and bilateral measurements. This could directly influence the conclusions. They may apply other methods including Generalized Estimating Equations.

We thank the reviewer for this comment. As suggested, I have updated the ‘Statistical analysis’ section with the following content (page10-11, line175−180）

To evaluate changes in CRT across three time points, repeated measures analysis of variance (ANOVA) was employed, followed by Tukey’s honest significant difference (HSD) test for post-hoc pairwise comparisons.

↓

`To evaluate changes in CRT across three time points, a linear mixed model was employed to account for the correlation between eyes of the same patient, followed by Tukey’s honest significant difference (HSD) test for post-hoc pairwise comparisons. `

Furthermore, we have replaced "repeated measures ANOVA" with "linear mixed model" throughout the RESULT section.

repeated measures ANOVA→ linear mixed model （page14, ine212）(page15, line221,231)(page16, line238)(page16, line254-255)(page17,line266）

Consequently, we observed no changes in the specific parameters that demonstrated statistical significance. For this reason, the summary of results in the abstract, which is based on the linear mixed model analysis, remains unchanged. However, as there were minor variations in the resulting P-values, we have updated the corresponding data in Figures 1, 2, and 3 accordingly.

The authors described EZ and ELM disruption was suppressed after switching to faricimab without statistical analysis.

We thank the reviewer for this comment. As suggested, Statistical analyses were performed to evaluate the integrity of the ellipsoid zone (EZ) and external limiting membrane (ELM) at three time points: one year before the switch, immediately before the switch, and one year after the switch. To account for the correlation between eyes and the influence of repeated measurements, a generalized linear mixed model (GLMM) was employed.

The results showed an overall trend toward stabilization or improvement in EZ and ELM integrity after switching to faricimab; however, these changes did not reach statistical significance (p > 0.05). We have updated the Methods and Results sections to include these statistical findings.

｀The disruption of the ellipsoid zone (EZ) and external limiting membrane (ELM) was analyzed using a generalized linear mixed model (GLMM).` (page11, line183-184)

`(P>0.05, linear mixed model［GLMM］)` (page16, line247)

Although the authors described treatment protocol after switching to faricimab, the one before switching is unclear. Did they treat with 1+PRN regimen along with the same retreatment criteria before switching? If so, please describe the reason why the treatment number increased even though faricimab was more effective than aflibercept or ranibizumab. In other words, there remain the possibility that intense treatment rather than switching drug achieved better macular edema control.

Thank you for your insightful comment. We received a similar comment from Reviewer 1.

As previously explained (please refer to our response to Reviewer 1), regarding treatment frequency, in cases that had reached a "treatment plateau" with conventional anti-VEGF therapy before the switch, follow-up intervals were clinically extended to several months because additional injections of the same agent no longer yielded significant anatomical improvements. However, following the switch to faricimab, we implemented a strict monthly follow-up protocol to accurately evaluate its efficacy and re-optimize the treatment.

Therefore, we consider that the increase in injection frequency was not simply a result of intensifying the existing therapy. Rather, it reflects a transition from a stage where conventional therapy had reached its functional limits to a period of active treatment reconstruction made possible by the introduction of faricimab. The fact that significant anatomical improvements were achieved despite the long-term refractory nature of these cases suggests the superior efficacy of faricimab in this specific patient population.

in addition to addressing the increase in the number of injections as part of our response to Reviewer 1 (pages 24, lines 386-406), we have added the following text to demonstrate that the therapeutic effects were achieved through faricimab itself, rather than simply through treatment intensification: (pages 25, lines 406-409)

"However, the observed anatomical improvements, despite the long-term refractory nature of the edema, suggest that the switch to faricimab provided a valuable clinical opportunity to reconstruct the treatment approach and move beyond the previous therapeutic limits."

Did authors consider diurnal variations in choroidal thickness measurements?

We thank the reviewer for this comment. To account for diurnal variation, all patients were examined at the diabetes clinic on the same day of the week (Thursdays) between 14:00 and 16:00. This information has been added to the Methods section.

Lines 431-
‘The treatment burden remained comparable to that pre-switching therapy’
Treatment number increased after switching to faricimab, didn’t it?

We thank the reviewer for this comment. As suggested, we had been changed that to ‘Although the burden of treatment has not been reduced compared to before the switching’ in the abstract (page29, line497-499).

The paragraph starting from line 382 is confusing. ‘Balanced vascular effect’ even though faricimab treatment reduced blood flow [26]??

We thank the reviewer for this comment. As suggested, we had deleted the following text ‘these observations suggest that faricimab may provide a balanced vascular effect that effectively reduces retinal edema while preserving choroidal structure.’(page26, line429-430) and has been changed‘these findings suggest that faricimab may effectively reduce retinal oedema by decreasing retinal blood flow, whilst preserving the choroidal vasculature.’ (page26, line431-433).

Figure 5
A and B does not include the hyperreflective band at the choroid-scleral interface in the choroidal thickness, whereas C includes it. This increases the choroidal thickness after faricimab injections and may influence the study results.

We thank the reviewer for this comment. As I had included the hyperreflective band at the choroid-sclera junction in my calculations, I have redrawn the diagram.
