# Supplementary material for: Real-world outcomes after switching to faricimab in treatment-resistant diabetic macular edema: A 1-year observational study with choroidal thickness assessment
Source: PLoS One. 2026 Jun 10;21(6):e0351168. doi: 10.1371/journal.pone.0351168 (PMC13252804; doi:10.1371/journal.pone.0351168)
Supplement: Peer review history

# Peer review: Decision Letter 0

**DOI:** 10.1371/journal.pone.0351168.r001

**Authors:** Daisuke Nagasato

6 Apr 2026

PONE-D-26-10259

Real-world outcomes after switching to faricimab in treatment-resistant diabetic macular edema: A 1-year observational study with choroidal thickness assessment

PLOS One

Dear Dr. Mori,

Thank you for submitting your manuscript to PLOS ONE. After careful consideration, we feel that it has merit but does not fully meet PLOS ONE’s publication criteria as it currently stands. Therefore, we invite you to submit a revised version of the manuscript that addresses the points raised during the review process.

Please submit your revised manuscript by May 21 2026 11:59PM. If you will need more time than this to complete your revisions, please reply to this message or contact the journal office at plosone@plos.org. When you're ready to submit your revision, log on to https://www.editorialmanager.com/pone/ and select the 'Submissions Needing Revision' folder to locate your manuscript file.

Please include the following items when submitting your revised manuscript:

- A letter that responds to each point raised by the academic editor and reviewer(s). You should upload this letter as a separate file labeled 'Response to Reviewers'.
- A marked-up copy of your manuscript that highlights changes made to the original version. You should upload this as a separate file labeled 'Revised Manuscript with Track Changes'.
- An unmarked version of your revised paper without tracked changes. You should upload this as a separate file labeled 'Manuscript'.

If you would like to make changes to your financial disclosure, please include your updated statement in your cover letter. Guidelines for resubmitting your figure files are available below the reviewer comments at the end of this letter.

If applicable, we recommend that you deposit your laboratory protocols in protocols.io to enhance the reproducibility of your results. Protocols.io assigns your protocol its own identifier (DOI) so that it can be cited independently in the future. For instructions see: https://journals.plos.org/plosone/s/submission-guidelines#loc-laboratory-protocols. Additionally, PLOS ONE offers an option for publishing peer-reviewed Lab Protocol articles, which describe protocols hosted on protocols.io. Read more information on sharing protocols at https://plos.org/protocols?utm_medium=editorial-email&utm_source=authorletters&utm_campaign=protocols.

As the corresponding author, your ORCID iD is verified in the submission system and will appear in the published article. PLOS supports the use of ORCID, and we encourage all coauthors to register for an ORCID iD and use it as well. Please encourage your coauthors to verify their ORCID iD within the submission system before final acceptance, as unverified ORCID iDs will not appear in the published article. *Only* the individual author can complete the verification step; PLOS staff *cannot* verify ORCID iDs on behalf of authors.

We look forward to receiving your revised manuscript.

Kind regards,

Daisuke Nagasato

Academic Editor

PLOS One

Journal Requirements:

When submitting your revision, we need you to address these additional requirements.

1. Please ensure that your manuscript meets PLOS ONE's style requirements, including those for file naming. The PLOS ONE style templates can be found at

https://journals.plos.org/plosone/s/file?id=wjVg/PLOSOne_formatting_sample_main_body.pdf and

https://journals.plos.org/plosone/s/file?id=ba62/PLOSOne_formatting_sample_title_authors_affiliations.pdf

2. We note that your Data Availability Statement is currently as follows: “If the data are all contained within the manuscript, enter the following: All relevant data are within the manuscript and its Supporting Information files.”

Please confirm at this time whether or not your submission contains all raw data required to replicate the results of your study. Authors must share the “minimal data set” for their submission. PLOS defines the minimal data set to consist of the data required to replicate all study findings reported in the article, as well as related metadata and methods (https://journals.plos.org/plosone/s/data-availability#loc-minimal-data-set-definition).

For example, authors should submit the following data:

- The values behind the means, standard deviations and other measures reported;

- The values used to build graphs;

- The points extracted from images for analysis.

Authors do not need to submit their entire data set if only a portion of the data was used in the reported study.

If your submission does not contain these data, please either upload them as Supporting Information files or deposit them to a stable, public repository and provide us with the relevant URLs, DOIs, or accession numbers. For a list of recommended repositories, please see https://journals.plos.org/plosone/s/recommended-repositories.

If there are ethical or legal restrictions on sharing a de-identified data set, please explain them in detail (e.g., data contain potentially sensitive information, data are owned by a third-party organization, etc.) and who has imposed them (e.g., an ethics committee). Please also provide contact information for a data access committee, ethics committee, or other institutional body to which data requests may be sent. If data are owned by a third party, please indicate how others may request data access.

3. If the reviewer comments include a recommendation to cite specific previously published works, please review and evaluate these publications to determine whether they are relevant and should be cited. There is no requirement to cite these works unless the editor has indicated otherwise.

[Note: HTML markup is below. Please do not edit.]

Reviewers' comments:

Reviewer's Responses to Questions

**Comments to the Author**

1. Is the manuscript technically sound, and do the data support the conclusions?

The manuscript must describe a technically sound piece of scientific research with data that supports the conclusions. Experiments must have been conducted rigorously, with appropriate controls, replication, and sample sizes. The conclusions must be drawn appropriately based on the data presented.

Reviewer #1: Yes

Reviewer #2: Partly

**********

2. Has the statistical analysis been performed appropriately and rigorously?

Reviewer #1: Yes

Reviewer #2: No

**********

3. Have the authors made all data underlying the findings in their manuscript fully available?

The PLOS Data policy requires authors to make all data underlying the findings described in their manuscript fully available without restriction, with rare exception (please refer to the Data Availability Statement in the manuscript PDF file). The data should be provided as part of the manuscript or its supporting information, or deposited to a public repository. For example, in addition to summary statistics, the data points behind means, medians and variance measures should be available. If there are restrictions on publicly sharing data—e.g. participant privacy or use of data from a third party—those must be specified.

Reviewer #1: No

Reviewer #2: Yes

**********

4. Is the manuscript presented in an intelligible fashion and written in standard English?

PLOS ONE does not copyedit accepted manuscripts, so the language in submitted articles must be clear, correct, and unambiguous. Any typographical or grammatical errors should be corrected at revision, so please note any specific errors here.

Reviewer #1: Yes

Reviewer #2: Yes

**********

5. Review Comments to the Author

Please use the space provided to explain your answers to the questions above. You may also include additional comments for the author, including concerns about dual publication, research ethics, or publication ethics. (Please upload your review as an attachment if it exceeds 20,000 characters)

Reviewer #1: Overall assessment

This is a valuable real-world report evaluating the clinical significance of a newer anti-VEGF agent for diabetic macular edema (DME), and the inclusion of choroidal thickness assessment is particularly interesting. If the key methodological and interpretive concerns below can be addressed through additional data/analyses and clearer descriptions, the manuscript could become a highly impactful contribution to the field.

Major comments

1) Data availability / transparency

The Data Availability statement indicates that “All relevant data are within the manuscript and its Supporting Information files.” However, as currently provided, I could not locate case-level/underlying data (e.g., per-eye CRT/CCT/BCVA values at each time point, injection dates/counts, or the dataset used for the analyses) in either the main text or the Supporting Information. It is possible that the relevant Supporting Information file(s) were not uploaded or were omitted during submission.

For transparency and reproducibility, please either (i) provide the underlying dataset as Supporting Information (de-identified, at least at the eye level), or (ii) revise the Data Availability statement to accurately reflect what is available and how the data can be accessed (e.g., upon reasonable request, subject to IRB restrictions), in accordance with journal policy.

2) Potential confounding by increased injection frequency (treatment burden not clearly “comparable”)

A central concern is that, although anatomical outcomes (e.g., CRT) improved after switching from aflibercept to faricimab, the injection frequency increased in the post-switch period. Therefore, it remains difficult to exclude the possibility that similar anatomical improvement might have been achieved simply by increasing injection frequency with aflibercept (i.e., an intensification effect rather than a switch effect). In this context, the Abstract statement “with a comparable treatment burden” appears overstated and should be revised or more carefully qualified.

3) Visit schedule under the “1+PRN” regimen and its relationship to injection frequency

The manuscript states that faricimab was administered using a “1+PRN” regimen, but it is unclear how often patients were actually reviewed (e.g., monthly vs. longer intervals). In real-world practice, follow-up intervals can vary substantially across institutions and even within the same center, depending on patient preferences and logistics. Please clarify the visit schedule after switching and, if applicable, before switching.

Specifically, I recommend reporting:

- The distribution of follow-up intervals (e.g., median and IQR, or a histogram) in the pre-switch and post-switch periods;

- Whether visit intervals changed after switching (and in what direction);

- The relationship between visit interval and injection frequency (e.g., correlation or another appropriate analysis).

If the follow-up interval shortened after switching, this could readily explain the increased injection frequency, and it would be important to account for this when interpreting the post-switch outcomes.

4) Interpretation of “worsening despite ongoing therapy” and risk of reverse causality / selection bias

In the Results, the manuscript states:

“Mean CRT increased from 398 ± 118 μm at 1 year before switching to 453 ± 112 μm immediately before switching, indicating progressive edema despite ongoing anti-VEGF therapy.”

Two points here:

(a) Placement and tone: The interpretive phrase (“indicating progressive edema…”) is more appropriate for the Discussion than the Results section, and the conclusion should be stated cautiously.

(b) Reverse causality / selection bias: In practice, switching often occurs because disease worsened or was perceived to be insufficiently controlled, rather than the worsening being evidence of inefficacy under a stable treatment strategy. In addition, because faricimab became available during the study period, it is unlikely that all eligible patients were switched immediately upon meeting a predefined retreatment threshold; patients with good control may be less likely to request/accept switching, and clinicians may be more inclined to recommend switching after demonstrating persistent/worsening edema. Thus, the observed increase in mean CRT immediately before switching may reflect “patients were switched when they worsened,” rather than “patients worsened despite therapy,” i.e., the direction of causality may be reversed.

To address this, please provide a clear explanation of:

- The explicit criteria and workflow used to decide when to switch (beyond the CRT threshold);

- Whether switching was triggered at a single visit with transient worsening versus sustained inadequate response across multiple visits;

- Any steps taken to mitigate selection bias.

Relatedly, the post-switch CRT reduction could partly reflect regression to the mean if switching tended to occur at a temporarily worse time point. Given the potential clinical impact of this type of real-world switch report on therapeutic adoption, careful framing in the Discussion (and an explicit statement in the Limitations) is warranted. While the data suggest potential benefit (including improvement compared with 1 year pre-switch), the possibility that part of the observed effect is explained by visit/injection intensity and/or regression to the mean should be transparently discussed.

5) Interpretation of CCT stability and outer retinal integrity changes

Several interpretive statements may also be influenced by the same confounding factors noted above.

- Regarding the sentence: “Notably, CCT remained stable during faricimab therapy despite a substantial reduction in retinal thickness, with no apparent relationship between the retinal and choroidal responses.” In real-world DME practice, switching decisions are typically driven by retinal findings (e.g., CRT/fluid), whereas CCT is not used clinically to guide switching. Therefore, the apparent dissociation between retinal and choroidal responses may be expected and does not necessarily support a mechanistic inference. Please consider tempering the causal/mechanistic interpretation and framing this as exploratory.

- Regarding outer retinal integrity: “Outer retinal disruption … increased from 28.6% to 35.7% … indicating progression … however … increased only slightly to 39.3% …” This pattern may also be at least partly explained by increased injection frequency and/or altered visit intervals after switching. Please consider addressing this possibility in the Discussion and/or Limitations, and avoid attributing suppression of progression to the switch unless supported by additional analyses.

Overall, if the authors can address the above issues—particularly by clarifying switching criteria, visit intervals, and the relationship between injection frequency and outcomes—many of these interpretive concerns could be substantially alleviated, and the manuscript would become a very important and clinically useful contribution.

Minor comment

The statement that real-world evidence “remains limited” is supported by the subsequent paragraph summarizing several switch studies; however, for readability, it may be helpful to add the key citations directly after this sentence (e.g., refs. 13–16).

Reviewer #2: The authors retrospectively investigated the visual outcome and OCT parameters before and after switching the anti-VEGF agents to faricimab in patients with refractory diabetic macular edema. This is an important theme in common practice. However, there are several issues that should be addressed.

I have a concern about statistical analyses. The authors included 28 eyes of 20 patients. Repeated measures ANOVA in its classical form cannot handle a mixed-eye dataset containing both unilateral and bilateral measurements. This could directly influence the conclusions. They may apply other methods including Generalized Estimating Equations.

The authors described EZ and ELM disruption was suppressed after switching to faricimab without statistical analysis.

Although the authors described treatment protocol after switching to faricimab, the one before switching is unclear. Did they treat with 1+PRN regimen along with the same retreatment criteria before switching? If so, please describe the reason why the treatment number increased even though faricimab was more effective than aflibercept or ranibizumab. In other words, there remain the possibility that intense treatment rather than switching drug achieved better macular edema control.

Did authors consider diurnal variations in choroidal thickness measurements?

Lines 431-

‘The treatment burden remained comparable to that pre-switching therapy’

Treatment number increased after switching to faricimab, didn’t it?

The paragraph starting from line 382 is confusing. ‘Balanced vascular effect’ even though faricimab treatment reduced blood flow [26]??

Figure 5

A and B does not include the hyperreflective band at the choroid-scleral interface in the choroidal thickness, whereas C includes it. This increases the choroidal thickness after faricimab injections, and may influence the study results.

**********

6. PLOS authors have the option to publish the peer review history of their article (what does this mean?). If published, this will include your full peer review and any attached files.

If you choose “no”, your identity will remain anonymous but your review may still be made public.

**Do you want your identity to be public for this peer review?** For information about this choice, including consent withdrawal, please see our Privacy Policy.

Reviewer #1: No

Reviewer #2: No

**********

[NOTE: If reviewer comments were submitted as an attachment file, they will be attached to this email and accessible via the submission site. Please log into your account, locate the manuscript record, and check for the action link "View Attachments". If this link does not appear, there are no attachment files.]

To ensure your figures meet our technical requirements, please review our figure guidelines: https://journals.plos.org/plosone/s/figures

You may also use PLOS’s free figure tool, NAAS, to help you prepare publication quality figures: https://journals.plos.org/plosone/s/figures#loc-tools-for-figure-preparation.

NAAS will assess whether your figures meet our technical requirements by comparing each figure against our figure specifications.

# Author comment: Author response to Decision Letter 1

**DOI:** 10.1371/journal.pone.0351168.r002

19 May 2026

We wish to express our appreciation to the editors and reviewers for their insightful comments, which have helped us significantly improve the paper. We have revised the manuscript (PONE-D-26-10259) according to the reviewer s’ comment. We respond for reviewers as attached file (Response to Reviewers).

We hope that our manuscript is now suitable for publication in PLOS one.

Attachment Submitted filename: Response to Reviewers.docx

# Peer review: Decision Letter 1

**DOI:** 10.1371/journal.pone.0351168.r003

**Authors:** Daisuke Nagasato

24 May 2026

Real-world outcomes after switching to faricimab in treatment-resistant diabetic macular edema: A 1-year observational study with choroidal thickness assessment

PONE-D-26-10259R1

Dear Dr. Mori,

We’re pleased to inform you that your manuscript has been judged scientifically suitable for publication and will be formally accepted for publication once it meets all outstanding technical requirements.

Within one week, you’ll receive an e-mail detailing the required amendments. When these have been addressed, you’ll receive a formal acceptance letter and your manuscript will be scheduled for publication.

An invoice will be generated when your article is formally accepted. Please note, if your institution has a publishing partnership with PLOS and your article meets the relevant criteria, all or part of your publication costs will be covered. Please make sure your user information is up-to-date by logging into Editorial Manager at Editorial Manager® and clicking the ‘Update My Information' link at the top of the page. For questions related to billing, please contact billing support.

If your institution or institutions have a press office, please notify them about your upcoming paper to help maximize its impact. If they’ll be preparing press materials, please inform our press team as soon as possible -- no later than 48 hours after receiving the formal acceptance. Your manuscript will remain under strict press embargo until 2 pm Eastern Time on the date of publication. For more information, please contact onepress@plos.org.

Kind regards,

Daisuke Nagasato

Academic Editor

PLOS One

Additional Editor Comments (optional):

Reviewers' comments:

Reviewer's Responses to Questions

**Comments to the Author**

1. If the authors have adequately addressed your comments raised in a previous round of review and you feel that this manuscript is now acceptable for publication, you may indicate that here to bypass the “Comments to the Author” section, enter your conflict of interest statement in the “Confidential to Editor” section, and submit your "Accept" recommendation.

Reviewer #1: All comments have been addressed

Reviewer #2: All comments have been addressed

**********

2. Is the manuscript technically sound, and do the data support the conclusions?

The manuscript must describe a technically sound piece of scientific research with data that supports the conclusions. Experiments must have been conducted rigorously, with appropriate controls, replication, and sample sizes. The conclusions must be drawn appropriately based on the data presented.

Reviewer #1: Yes

Reviewer #2: (No Response)

**********

3. Has the statistical analysis been performed appropriately and rigorously?

Reviewer #1: Yes

Reviewer #2: (No Response)

**********

4. Have the authors made all data underlying the findings in their manuscript fully available?

The PLOS Data policy requires authors to make all data underlying the findings described in their manuscript fully available without restriction, with rare exception (please refer to the Data Availability Statement in the manuscript PDF file). The data should be provided as part of the manuscript or its supporting information, or deposited to a public repository. For example, in addition to summary statistics, the data points behind means, medians and variance measures should be available. If there are restrictions on publicly sharing data—e.g. participant privacy or use of data from a third party—those must be specified.

Reviewer #1: Yes

Reviewer #2: (No Response)

**********

5. Is the manuscript presented in an intelligible fashion and written in standard English?

PLOS ONE does not copyedit accepted manuscripts, so the language in submitted articles must be clear, correct, and unambiguous. Any typographical or grammatical errors should be corrected at revision, so please note any specific errors here.

Reviewer #1: Yes

Reviewer #2: (No Response)

**********

6. Review Comments to the Author

Please use the space provided to explain your answers to the questions above. You may also include additional comments for the author, including concerns about dual publication, research ethics, or publication ethics. (Please upload your review as an attachment if it exceeds 20,000 characters)

Reviewer #1: Thank you for the thorough revision and detailed responses. I have reviewed the revised manuscript and the point-by-point replies, and I am satisfied that the concerns raised during peer review have been appropriately addressed and clearly incorporated into the manuscript. I have no further comments.

Reviewer #2: (No Response)

**********

7. PLOS authors have the option to publish the peer review history of their article (what does this mean?). If published, this will include your full peer review and any attached files.

If you choose “no”, your identity will remain anonymous but your review may still be made public.

**Do you want your identity to be public for this peer review?** For information about this choice, including consent withdrawal, please see our Privacy Policy.

Reviewer #1: **Yes:** Hidenori Takahashi

Reviewer #2: No

**********

# Editor report: Acceptance letter

**DOI:** 10.1371/journal.pone.0351168.r004

**Authors:** Daisuke Nagasato

PONE-D-26-10259R1

PLOS One

Dear Dr. Mori,

I'm pleased to inform you that your manuscript has been deemed suitable for publication in PLOS One. Congratulations! Your manuscript is now being handed over to our production team.

At this stage, our production department will prepare your paper for publication. This includes ensuring the following:

* All references, tables, and figures are properly cited

* All relevant supporting information is included in the manuscript submission,

* There are no issues that prevent the paper from being properly typeset

You will receive further instructions from the production team, including instructions on how to review your proof when it is ready. Please keep in mind that we are working through a large volume of accepted articles, so please give us a few days to review your paper and let you know the next and final steps.

Lastly, if your institution or institutions have a press office, please let them know about your upcoming paper now to help maximize its impact. If they'll be preparing press materials, please inform our press team within the next 48 hours. Your manuscript will remain under strict press embargo until 2 pm Eastern Time on the date of publication. For more information, please contact onepress@plos.org.

You will receive an invoice from PLOS for your publication fee after your manuscript has reached the completed accept phase. If you receive an email requesting payment before acceptance or for any other service, this may be a phishing scheme. Learn how to identify phishing emails and protect your accounts at https://explore.plos.org/phishing.

If we can help with anything else, please email us at customercare@plos.org.

Thank you for submitting your work to PLOS ONE and supporting open access.

Kind regards,

PLOS ONE Editorial Office Staff

on behalf of

Dr. Daisuke Nagasato

Academic Editor

PLOS One
